# Identification of the Appropriate Boundary Size to Use When Measuring the Food Retail Environment Surrounding Schools

**DOI:** 10.3390/ijerph9082715

**Published:** 2012-07-31

**Authors:** Laura Seliske, William Pickett, Andrei Rosu, Ian Janssen

**Affiliations:** 1 Department of Community Health and Epidemiology, Queen’s University, Kingston, ON K7L 3N6, Canada; Email: lseliske@gmail.com (L.S.); will.pickett@queensu.ca (W.P.); 2 Clinical Research Center, Angada 3, Kingston General Hospital, 76 Stuart St., Kingston, ON K7L 2V7, Canada; 3 School of Kinesiology and Health Studies, Queen’s University, 28 Division St., Kingston, ON K7L 3N6, Canada; Email: rosua@queensu.ca (A.R.)

**Keywords:** built environment, youth, schools, geographic information systems

## Abstract

This study included 6,971 students in grades 9 and 10 (ages 13 to 16 years) from 158schools who participated in the 2009/2010 Health Behaviour in School-aged Children Study. Students provided information on where they typically ate lunch. The number of food retailers was obtained for six road network buffer sizes (500, 750, 1,000, 1,500, 2,000, and 5,000 meters) surrounding schools. Associations between the presence of food retailers near schools and students’ lunchtime eating behaviours were examined using multilevel logistic regression. Comparisons of model fit statistics indicated that the 1,000 m buffer provided the best fit. At this distance, students with ≥3 food retailers near their schools had a 3.42 times greater relative odds (95% CI: 2.12–5.52) of eating their lunchtime meal at a food retailer compared to students with no nearby food retailers. Students who had ≥2 food retailers within 750 m of their schools had a 2.74 times greater relative odds (95% CI: 1.75–4.29), while those who had ≥1 food retailer within 500 m of their schools had 2.27 times greater relative odds of eating at food retailer (95% CI: 1.46–3.52) compared to those with no nearby food retailers. For distances greater than 1,000 m, no consistent relationships were found.

## 1. Introduction

Over the past three decades, the prevalence of obesity among youth has increased dramatically worldwide [1]. There has been a lack of long-term success in its prevention via interventions that focus solely on individual-level factors [2]. This has resulted in the adoption of a more comprehensive understanding of its determinants, which include the influence of the food retail environment on eating behaviours in young people [3]. The food retail environment refers to the availability of food retailers such as fast food restaurants and convenience stores. In general, foods sold at these retailers are of poor nutritional quality [4,5,6] and excess consumption is associated with adverse health outcomes such as obesity and cardiometabolic diseases [7,8,9,10]. For young people, the food retail environment includes food retailers surrounding their homes and schools [11,12]. Relationships between the food retail environment and students’ lunchtime eating behaviours [13] and obesity [14,15,16] have been found.

The food retail environment has been measured using both subjective [17,18] and objective [19,20] methods. Subjective measures rely on perceptions about the self-reported availability of nearby food retailers, while objective measures use geographic information systems (GIS) software to map food retailers within a given geographic area. Due to the poor correlation between subjective and objective measures of the food retail environment [21], objective GIS-based measures are preferred. GIS-based studies have used a variety of boundary types to capture the food retail environment, including census boundaries [22], ZIP codes [16], circular buffers [14], and road network buffers [20]. A limitation of census and ZIP code boundaries are that they were designed for administrative purposes and vary in size. Circular buffers overcome this limitation, but do not reflect how people travel from one location to another. Furthermore, road network-based food retailer measures are more strongly related to young peoples’ eating behaviours compared to circular buffer based measures [13].

Recent literature reviews have identified the selection of the appropriate buffer size as a key methodological issue for studies of the food retail environment [3,23]. Some studies have addressed this by using various buffer sizes [14,20,24,25]. Davis and Carpenter [14] found that chain fast food restaurants within 0 to 400 m and 400 m to 800 m of schools, but not 800 m to 1,200 m, were associated with students’ body mass index (BMI) values. Laska *et al.*, [24] found that food retailers within 1,600 m of homes, but not 800 m or 3,000 m, were associated with adolescents’ sugar sweetened beverage intake and obesity. However, none of the studies conducted a formal analysis to identify which buffer size was the best predictor of eating behaviours or obesity. Furthermore, some studies only used two buffer sizes [20,24,25], which may have resulted in the most appropriate buffer size being excluded. Finally, some studies took place within a single city [24,25] or state [14,20], limiting their representativeness.

The primary objective of this study was to identify the most appropriate buffer size to use when studying the relationship between the school food retail environment and the eating behaviours of Canadian students. To achieve this, the food retail environment surrounding schools was measured using several buffer sizes. Multilevel logistic regression was used to examine associations between the number of food retailers in each buffer size and students’ lunchtime eating behaviours. Model fit statistics were used to determine the most appropriate buffer size.

## 2. Experimental Section

### 2.1. Overview of Study Design

This was a multilevel cross-sectional analysis of schools participating in the 2009/2010 Canadian Health Behaviour in School-aged Children (HBSC) study. Addresses of food retailers near schools were obtained using an online food retailer database. The number of food retailers surrounding each school was obtained for the following road network-based buffers: 500 m, 750 m, 1,000 m, 1,500 m, 2,000 m and 5,000 m. Associations between the presence of food retailers and students’ reports of eating their lunch at a food retailer were then assessed.

### 2.2. Study Sample

The 2009/2010 Canadian HBSC study involved a survey of students from 436 schools and collected information on a variety of health behaviours in students in grades 6 to 10 (approximate ages 11–16 years). Classes were the primary sampling unit, and they were stratified by province, with an oversampling of some provinces and the three northern territories. Two Canadian provinces with small populations (New Brunswick and Prince Edward Island) were unable to participate in the 2009/2010 survey. The HBSC also excludes students in private schools, incarcerated youth, special needs schools and students who are home schooled. Ethics approval was obtained from the Queen’s University Health Sciences Research Ethics Board. Subject consent was obtained at the school board and school levels as well as from parents or guardians (either explicitly or implicitly, as determined by school board policy).

In the current analysis, the sample was restricted to the 169 schools where students were permitted to leave school grounds, making it possible for them to access nearby food retailers. Because only 1.1% of grade 6 to 8 students reported the study outcome, they were excluded from the analysis. Due to missing information on food sources within schools or in the school neighbourhoods, a further 11 schools were excluded. Finally, 940 students were excluded because of missing data on key variables. The final analyses involved 6,971 students from 158 schools.

### 2.3. School Food Retail Environment

**Figure 1:**
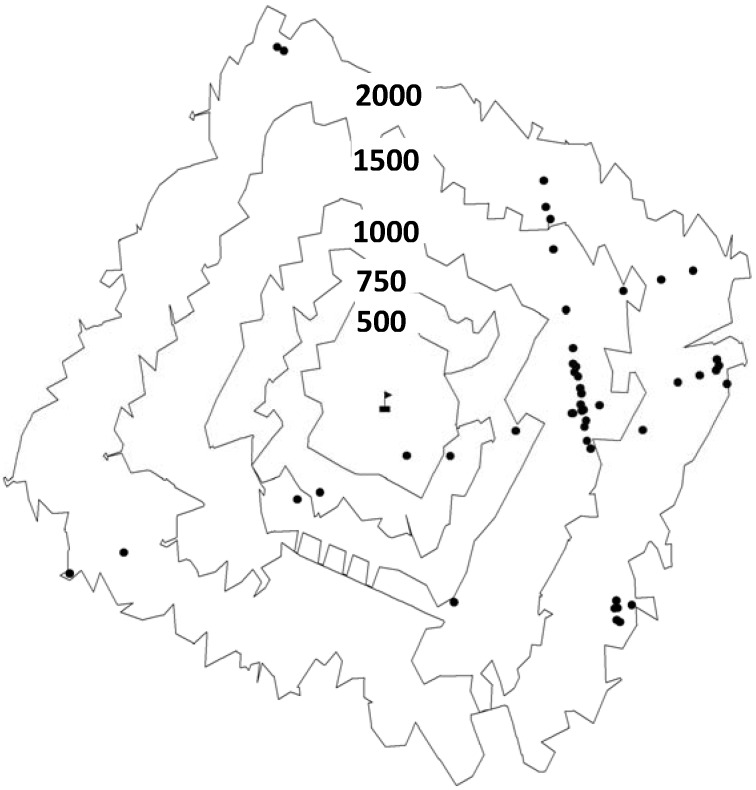
The school is in the centre of the figure and is surrounded by increasing buffer sizes, ranging from 500 m to 2,000 m. The food retailers are represented by black circles.

The addresses of the 158 eligible HBSC schools were mapped in ArcGIS (ESRI, version 9.3) and road network-based buffers were constructed using the following distances: 500 m, 750 m, 1,000 m, 1,500 m, 2,000 m and 5,000 m. These distances were selected based upon existing precedents [19,20,26,27,28]. Road network-based buffers were chosen instead of circular buffers because they are a better measure of the food retail environment surrounding schools [13]. The buffers were created using a road network database provided by CanMaps Streetfiles (DMTI Spatial Inc., v.2009.4, Markham ON, Canada). Roads extending outwards from the schools were followed until they reached their specified endpoint. Lines connecting the endpoints were used to create the border of the road network-based buffers. Figure 1 provides an illustration of the multiple road network buffers used in this study.

Convenience stores, fast food restaurants, and coffee/donut shops were used to develop the primary independent variable of interest. These food retailers directly corresponded to the lunchtime eating question used to create the study outcome. Addresses of these food retailers were obtained using an online Yellow Pages directory (www.yellowpages.ca). The Yellow Pages directory was chosen because it provided the most accurate information available on food retailer locations [29]. School addresses were entered into the Yellow Pages directory. The search term ‘convenience stores’ was used to obtain convenience store addresses. There was no single search term to use for fast food restaurants and coffee/donut shops because many of them were listed under the full service restaurant category. Therefore, we searched for the top chain food retailers, as has been done in previous studies [28,30]. The top 75% of 200 chain food retailers in Canada in 2009 were obtained from Technomic Inc. [31], and are available from the authors upon request.

All food retailers within the road network buffers were mapped using ArcGIS software. The mapping procedure in ArcGIS provided a score evaluating the accuracy of each mapped location. For food retailers whose street addresses had a score of less than 80%, the Street View tool in Google Earth (©2011 Google) was used to confirm the location and obtain latitude and longitude coordinates to map them manually in ArcGIS. Because the number of food retailers within the buffers was positively skewed, they were categorized into groups. All categories were based on thresholds established for the 500 m network buffer size. At this distance, 74% of schools had no nearby food retailers (categorized as “none”), and the remaining 26% were categorized as having “1 or more” food retailers. For the remaining buffer sizes, a “none” category was created, and a threshold of the top ~26% was used to denote the category with the highest number of food retailers. The thresholds for the remaining categories were created to ensure an approximately even distribution of schools across the categories and similar sizes among the categories.

### 2.4. Lunchtime Eating Outcome

The outcome of this study was obtained from the response to the following question: “*Where* *do* *you* *usually* *eat* *your* *lunch* *or* *mid-day* *meal* *on* *school* *days?*” Students who chose the response “*snack-bar*, *fast* *food* *restaurant*, *café*” were considered to regularly purchase their lunch from food retailers. Those who chose the remaining responses (“*at* *school*”, “*at* *home*”, “*at* *someone* *else’s* *house*”, “*do* *not* *eat* *lunch/mid-day* *meal*”, or “*other*”) were classified as those who did not typically obtain their lunch from food retailers.

### 2.5. Confounders

Individual-level variables, including age, sex, and socioeconomic status were considered as potential confounders since fast food consumption varies by these characteristics [6,32,33]. To obtain information on socioeconomic status, the HBSC uses the previously validated family affluence scale (FAS) [34]. Because cafeterias, vending machines, and school snack shops are associated with students’ eating behaviours [35,36,37], they were considered as potential school-level confounders. The distance between students’ homes and schools was not included as a confounder because it was not related to the study outcome with a subsample of urban youth who reported their postal code in the survey (data not shown).

### 2.6. Analysis

All analyses were conducted using SAS statistical software, version 9.2 (SAS Institute, Cary, NC, USA). Multilevel logistic regression was carried out to examine the relationship between the presence of food retailers near schools (convenience stores, fast food restaurants, and coffee/donut shops) and the likelihood of students eating their lunch at these food retailers using different sized road network buffers. For each buffer size, the multivariate model building process began with the introduction of the individual-level confounders and proceeded using a backwards elimination approach. Next, the school-level food exposure variables were forced into the model because we were interested in accounting for food sources within school as well as those surrounding schools. 

The Akaike information criterion (AIC), which is a measure of goodness-of-fit when comparing two or more regression models, was determined for the final multivariate models. A difference in AIC values of between 2 to 7 indicates a moderate difference in fit of the models, while a difference of 7 or more indicates a large difference in model fit [38]. Using the difference in AIC values compared to the lowest AIC value, the relative likelihoods were determined. Next, the Akaike’s weight was calculated for each model, which is the relative likelihood for each model divided by the total relative likelihood for all candidate models, and is expressed as a percentage. This percentage indicates the probability that a regression model is the best choice among a set of candidate models based on model fit [38]

## 3. Results

The median number of student respondents per school was 37 (interquartile range: 23–55). Table 1 shows the school- and student-level characteristics. Nearly 40% of the schools were located in large metropolitan centres and 59.5% were secondary schools. There was an approximately equal distribution of males and females and only 8.0% of the study sample was in the lowest family affluence group. Of the respondents who self-reported their height and weight, 19.6% were overweight or obese according to the International Obesity Task Force body mass index criteria [39]. Two thirds of the sample typically ate their lunch at school, 15.2% typically ate their lunch at home, and 7.4% typically ate their lunch in a snack bar, fast food restaurant, or cafe. Over three quarters of the schools had a cafeteria, and nearly two thirds had vending machines selling sugared drinks. Less than half of the schools had vending machines that sold milk or candy and potato chips, while less than a third had a tuck shop/snack bar.

**Table 1:** Characteristics of the school sample from the 2009/2010 HBSC.

			N	%
**School-level variables**				
****	**School type**			
		Secondary (grades 9–12)	94	59.5
		Mixed	64	40.5
**Urban rural status**				
		Large urban centre (≥100,000 people)	62	39.2
		Medium urban centre (20,000–99,999)	15	9.5
		Small urban centre (1,000–19,000)	38	24.1
		Rural (<1,000)	43	27.2
**Food Sources within Schools**				
		Cafeteria	120	76.0
		Sugared drinks vending machines	97	61.4
		Milk vending machines	75	47.5
		Candy and potato chip vending machines	64	40.5
		School tuck shop/snack-bar	51	32.3
**Individual-level variables**				
****	**Sex**			
		Male	3,381	48.5
		Female	3,590	51.5
****	**Age (years)**			
		13	33	0.5
		14	2,339	33.6
		15	3,280	47.1
		≥16	1,319	18.9
****	**Family affluence scale**			
		Low	560	8.0
		Moderate	2,531	36.3
		High	3,880	55.7
****	**Where students eat mid-day meal**			
		At school	4,719	67.7
		At home	1,056	15.2
		In a snack bar, fast food restaurant or café	517	7.4
		Never eat a midday meal	307	4.4
		Somewhere else	209	3.0
		At someone else’s home	163	2.3
****	**Weight status**			
		Non-overweight	4,823	69.2
		Overweight	1,018	14.6
		Obese	346	5.0
		Missing data	784	11.3

Table 2 provides the quantity of food retailers within the various buffer sizes. Very few food retailers were obtained within the smallest buffers; only 88 food retailers were located within 500 m buffers for the 158 schools in this study. The median number of food retailers ranged from 0 to 13 across the buffer sizes, and median values were greater than zero for buffers of 1,000 m and larger.

**Table 2:** The distribution of food retailers within the various buffer sizes.

Buffer size	Total number of	25th	50th	75th	Maximum
	food retailers	Percentile	Percentile	Percentile	
500 m	88	0	0	1	7
750 m	193	0	0	2	9
1,000 m	349	0	1	3	15
1,500 m	768	0	3	7	27
2,000 m	1,279	1	6	12	53
5,000 m	4,798	1	13	43	275

Results of the multilevel logistic regression are shown in Table 3. Model 1 shows the bivariate relationships, Model 2 controlled for individual-level covariates, and Model 3 controlled for individual- and school-level covariates. The strongest relationship appeared to be for the 1,000 m buffer, where students who had 3 or more food retailers within 1,000 m of their schools had 3.42 (95% CI: 2.12–5.52) times the relative odds of eating lunch at a food retailer compared to students with no food retailers within 1,000 m. The smallest two buffer sizes also showed relationships with lunchtime eating behaviours. At buffer sizes greater than 1,000 m, these relationships were weaker and not statistically significant (exceptions: 7–10 and 11 or more food retailers for the 2,000 m buffer).

The AIC-related values comparing goodness-of-fit between the final regression models (Model 3 in Table 3) for the different buffer sizes are shown in Table 4. The 1,000 m buffer had the lowest AIC value. The difference in AIC values between the 1,000 m buffer model and all remaining models was greater than the threshold value of 7, which indicated that there was a substantially better model fit for the 1,000 m buffer. Results from the Akaike’s weights showed there was a 98.8% probability that the 1,000 m road network buffer provided the best fit among the candidate models.

**Table 3:** Food retail buffer size and eating lunch at a snack-bar, fast food restaurant, or café.

Number of food retailers within buffer		Number of schools (%)	Odds ratio (95% confidence interval)		
			Model 1	Model 2	Model 3
**500 m**					
	None	117 (74.1)	1.00	1.00	1.00
	1 or more	41 (25.9)	2.15 (1.38–3.36)	2.20 (1.40–3.46)	2.27 (1.46–3.52)
**750 m** ****					
	None	89 (56.3)	1.00	1.00	1.00
	1	26 (16.5)	1.44 (0.82–2.54)	1.50 (0.84–2.66)	1.40 (0.79–2.48)
	2 or more	43 (27.2)	2.84 (1.81–4.47)	2.90 (1.83–4.60)	2.74 (1.75–4.29)
**1,000 m**					
	None	62 (39.2)	1.00	1.00	1.00
	1–2	51 (32.3)	1.24 (0.76–2.02)	1.25 (0.76–2.06)	1.20 (0.74–1.95)
	3 or more	45 (28.4)	3.49 (2.17–5.61)	3.55 (2.19–5.76)	3.42 (2.12–5.52)
**1,500 m**					
	None	43 (27.2)	1.00	1.00	1.00
	1–2	25 (15.8)	1.21 (0.60–2.45)	1.20 (0.59–2.45)	1.22 (0.59–2.53)
	3–4	20 (12.7)	1.45 (0.71–2.97)	1.43 (0.69–2.97)	1.37 (0.66–2.88)
	5–6	26 (16.5)	1.88 (0.97–3.64)	1.91 (0.98–3.74)	1.85 (0.94–3.65)
	7 or more	44 (27.8)	3.06 (1.72–5.44)	3.13 (1.75–5.62)	2.96 (0.64–5.34)
**2,000 m**					
	None	34 (21.5)	1.00	1.00	1.00
	1–3	28 (17.7)	1.34 (0.64–2.83)	1.32 (0.62–2.82)	1.38 (0.65–2.96)
	4–6	25 (15.8)	1.50 (0.71–3.19)	1.54 (0.72–3.30)	1.50 (0.67–3.34)
	7–10	22 (13.9)	2.37 (1.16–4.87)	2.43 (1.17–5.04)	2.28 (1.07–4.86)
	11 or more	45 (28.5)	2.56 (1.33–4.93)	2.57 (1.32–5.02)	2.48 (1.23–5.02)
**5,000 m**					
	None	30 (19.0)	1.00	1.00	1.00
	1–9	35 (22.2)	1.39 (0.65–2.95)	1.38 (0.64–2.96)	1.26 (0.58–2.77)
	11–19	31 (19.6)	1.65 (0.77–3.51)	1.69 (0.78–2.65)	1.48 (0.66–3.33)
	20–29	11 (7.0)	2.04 (0.75–5.57)	2.01 (0.72–5.57)	1.94 (0.67–5.61)
	30–39	11 (7.0)	2.22 (0.82–6.00)	2.18 (0.79–6.01)	1.95 (0.70–5.45)
	40 or more	40 (25.3)	2.09 (1.02–4.29)	2.11 (1.02–4.38)	1.81 (0.83–3.97)

## 4. Discussion

This study identified the 1,000 m road network buffer as the appropriate buffer size for examining the relationship between the school food retail environment and lunchtime eating behaviours. Based upon an average walking speed of 4–5 km/hour in adolescents [40], this is a distance that could be walked in approximately 10–15 minutes. At distances less than 1,000 m, less than half of the schools had at least one food retailer present, suggesting these buffers were too small to capture a sufficient number of food retailers. Distances greater than 1,000 m may be perceived by students as being too far for them to travel during the time allotted for their lunch break. In Canada, lunch breaks at school typically range from 30 minutes to 1 hour in length, with most lasting about 45 minutes. If it takes 10 minutes to walk 1 km, there would be sufficient time to purchase and eat lunch at a fast food or convenience retailer located within 1,000 m of schools.

**Table 4:** Comparison of AIC values, relative likelihoods, and Akaike’s weights across various buffer sizes.

Buffer size	AIC value	Δ AIC *vs.* 1,000 m distance	Relative likelihood	Akaike’s weight (%)
			e ^(−0.5 × ^^Δ^ ^AIC )^	
500 m	3,356.64	12.77	0.00169	0.167
750 m	3,353.12	9.25	0.00980	0.969
1,000 m	3,343.87	0	1.00000	98.836
1,500 m	3,360.21	16.34	0.00028	0.028
2,000 m	3,366.72	22.85	0.00001	0.000
5,000 m	3,373.94	30.07	0.00000	0.000
**Total**			1.01178	100.00

The association between the number of food retailers within 500 m, 750 m, and 1,000 m road network buffers around schools and students’ eating behaviours was consistent with observations by Davis and Carpenter [14]. They found that chain fast food restaurants within 0 to 400 m and 400 m to 800 m of schools were associated with students’ BMI values. An advantage of our study is that it used model fit criteria to determine that the 1,000 m buffer size was the most appropriate. It is important to note that the most appropriate buffer size may be different for non-school settings. Studies of the food retail environment surrounding homes have provided mixed results, with two suggesting that a relatively small (*i.e.*, 400 to 800 m) buffer size may be the most appropriate [20,25], and one study suggesting that larger (*i.e.*, 1,600 m) buffer sizes are most appropriate [24].

Results from this study provide important evidence regarding the appropriate buffer size to use when measuring the food retail environment surrounding schools. Namely, using a buffer that is either too large or too small may obscure relationships with eating behaviours and result in weakened study findings. Furthermore, having a standard buffer size will enhance comparability across studies. Finally, results from studies such as this one are needed to inform the implementation and evaluation of policies aimed at optimizing the built environment surrounding schools. Specifically, our findings suggest that policies that limit the number of food retailers around schools should consider a 1,000 m distance and a maximum of 2 fast food restaurants, coffee/donut shops, and/or convenience stores. More than one quarter (28.4%) of the schools studied here would be affected by such a policy.

This study has some key strengths including: the large and geographically diverse study sample, the wide variety of buffer sizes, and the use of goodness-of-fit statistics to assess the relative contribution of each buffer size to model fit. Another key strength of this study is that it considered constraints on mobility and time when measuring accessibility to food retailers. In other words, by limiting our study outcome to a specific time and place to eat (e.g., lunch time during the school day), we accounted for these limitations on spatial accessibility. Measures of spatial accessibility that consider limitations imposed by time and space are weakly correlated with those which do not consider them [41] and this has been confirmed in studies of the Canadian food environment [42]. Future studies should consider food environments and eating behaviours that are specific to them, such as the journey to and from school as well as the home environment.

A key limitation of this study was that the GIS database used to obtain information on the food environment does not provide a completely accurate measure of the food environment. Furthermore, for practical reasons, only the top chain fast food restaurants and donut/coffee retailers were included in the food retail environment measures. In addition, the survey data were obtained by self-report and this may introduce bias due to the social desirability of eating healthy foods [43]. It is also possible that students may have reported higher food intakes than what they actually consumed, since young people who have low body weights tend to over-report their energy intake [44]. Finally, the cross-sectional nature of this study made it impossible to assess temporality between the presence of food retailers and eating behaviours. However, it is unlikely that students chose to attend schools based on the presence of nearby food retailers. In fact, there is evidence to suggest that fast food restaurants cluster around schools [11], indicating that some food retailers may be preferentially located near schools. 

## 5. Conclusions

The results from this study indicated that the most appropriate road network buffer distance when assessing the food retail environment surrounding Canadian schools was 1,000 m. Future studies investigating the school food retail environment should consider using street network buffers of this size. Having a consistent buffer size across studies will not only help inform policies and interventions directed at the modification of the food environment surrounding schools, but will also help facilitate comparisons across studies. 
